# *In-vitro* Characterization of a Hernia Mesh Featuring a Nanostructured Coating

**DOI:** 10.3389/fbioe.2020.589223

**Published:** 2021-01-20

**Authors:** Giulia Giuntoli, Giuliana Muzio, Chiara Actis, Alessandro Ganora, Stefano Calzone, Matteo Bruno, Gianluca Ciardelli, Irene Carmagnola, Chiara Tonda-Turo

**Affiliations:** ^1^Department of Mechanical and Aerospace Engineering, Politecnico di Torino, Turin, Italy; ^2^POLITO BIOMedLAB, Politecnico di Torino, Turin, Italy; ^3^Department of Clinical and Biological Sciences, University of Turin, Turin, Italy; ^4^Dipromed Medical Devices S.r.l, Turin, Italy; ^5^Department for Materials and Devices of the National Research Council, Institute for the Chemical and Physical Processes (CNR-IPCF UOS), Pisa, Italy

**Keywords:** polypropylene mesh, abdominal hernia repair, multicomponent device, nanostructured coating, nanofibers

## Abstract

Abdominal hernia repair is a frequently performed surgical procedure worldwide. Currently, the use of polypropylene (PP) surgical meshes for the repair of abdominal hernias constitutes the primary surgical approach, being widely accepted as superior to primary suture repair. Surgical meshes act as a reinforcement for the weakened or damaged tissues and support tissue restoration. However, implanted meshes could suffer from poor integration with the surrounding tissues. In this context, the present study describes the preliminary evaluation of a PCL-Gel-based nanofibrous coating as an element to develop a multicomponent hernia mesh device (meshPCL-Gel) that could overcome this limitation thanks to the presence of a nanostructured biomimetic substrate for enhanced cell attachment and new tissue formation. Through the electrospinning technique, a commercial PP hernia mesh was coated with a nanofibrous membrane from a polycaprolactone (PCL) and gelatin (Gel) blend (PCL-Gel). Resulting PCL-Gel nanofibers were homogeneous and defect-free, with an average diameter of 0.15 ± 0.04 μm. The presence of Gel decreased PCL hydrophobicity, so that membranes average water contact angle dropped from 138.9 ± 1.1° (PCL) to 99.9 ± 21.6°, while it slightly influenced mechanical properties, which remained comparable to those of PCL (*E* = 15.7 ± 2.7 MPa, σ_*R*_= 7.7 ± 0.6 ε_*R*_ = 118.8 ± 13.2%). Hydrolytic and enzymatic degradation was conducted on PCL-Gel up to 28 days, with maximum weight losses around 20 and 40%, respectively. The meshPCL-Gel device was obtained with few simple steps, with no influences on the original mechanical properties of the bare mesh, and good stability under physiological conditions. The biocompatibility of meshPCL-Gel was assessed by culturing BJ human fibroblasts on the device, up to 7 days. After 24 h, cells adhered to the nanofibrous substrate, and after 72 h their metabolic activity was about 70% with respect to control cells. The absence of detectable lactate dehydrogenase in the culture medium indicated that no necrosis induction occurred. Hence, the developed nanostructured coating provided the meshPCL-Gel device with chemical and topographical cues similar to the native extracellular matrix ones, that could be exploited for enhancing the biological response and, consequently, mesh integration, in abdominal wall hernia repair.

## Introduction

Abdominal hernia repair is one of the most common surgical procedure worldwide (Eurostat, 2016). A hernia is defined as a protrusion of a tissue or organ from the cavity where it is normally contained, and it can be either congenital or developed over time. Generally, this type of hernia occurs at an area of weakness in the abdominal wall, and often develops at the site of previous surgical incisions (incisional hernia) (Le Huu Nho et al., 2012).

In hernia repair, the use of prosthetic meshes is now largely accepted as gold standard of the surgical strategy. Indeed, the mesh guarantees proper reinforcement of the weakened area and promotes tissue restoration, while diminishing the risks of recurrence when compared to other techniques, such as suture repair (Burger et al., 2004; Kokotovic et al., 2016).

However, there are still several post-operative complications affecting a large number of patients, such as general discomfort, movement restriction, chronic pain (Fränneby et al., 2006), infections (Hawn et al., 2011), fibrosis, adhesions (Dinsmore et al., 2000), or erosion, which can require a revision surgery or even the complete removal of the prosthesis (Kokotovic et al., 2016), mainly in the long-term (Burger et al., 2004).

Mesh design is a crucial point to define the properties and surgical outcomes of the implant, thus, it should fulfill a number of requirements (Rastegarpour et al., 2016). The choice of mesh materials and manufacturing methods will influence mesh biocompatibility, mechanical properties (Est et al., 2017), porosity (Klinge et al., 2002), and degradation rates (Jangö et al., 2017). Meshes could be woven or knitted, with different yarn patterns and filament size, number, and density. Due to the high number of variables involved, there are a multitude of available commercial products in the market (Brown and Finch, 2010; Coda et al., 2012; Rastegarpour et al., 2016; Deeken and Lake, 2017; Sanbhal et al., 2018). It is now widely accepted that knitted monofilament meshes have reduced inflammation when compared to multifilament meshes, and that light-weight highly porous meshes, with pores larger than 75 μm, have more flexibility, enhanced tissue ingrowth, and reduced scar formation with respect to heavy-weight multifilament meshes (Klosterhalfen et al., 2005). Mechanical properties are another crucial parameter for the success of the implant and should closely match physiological values to restore the abdominal wall functions (Taylor, 2018). An excessively rigid mesh could cause tissues damage, whereas a mesh that is too soft lacks strength for tissue reinforcement, leading to hernia recurrence (Simón-Allué et al., 2018).

Since its first use in the late 50's, the gold standard material of hernia meshes is polypropylene (PP) (Usher et al., 1958). Permanent synthetic PP meshes have many desirable advantages since PP is biocompatible, hydrophobic, physically inert, non-immunogenic, and non-cancerogenic. However, its use is not completely free from adverse reactions. Literature reports that PP meshes often adhere with the underlying contacting viscera (Dinsmore et al., 2000), or that they cause a chronic state of inflammation which hampers tissue integration (Klinge et al., 2002; Brown et al., 2015), although performing better in terms of host response when compared to other materials (Michelle et al., 2017).

Therefore, composite meshes have been developed as more performant solutions (Deeken et al., 2011; Sanbhal et al., 2018) with the aim to reduce post-operative complications by preventing visceral adhesion, fibrosis and bacterial infections, while promoting healthy tissue integration thanks to increased biocompatibility and better tissue ingrowth. For instance, meshes coated with degradable (Takaoka et al., 2009; Faulk et al., 2014; Wolf et al., 2014) and non-degradable (Poppas et al., 2016) hydrogels resulted in mitigated inflammatory and foreign body reaction (FBR) responses. Similarly, antibacterial coatings, which act locally at the site of the implant, were shown to reduce or prevent the risk of mesh-related infections both *in vitro* and *in vivo* (Harth et al., 2010), avoiding the need of systemic drug administration (Aydinuraz, 2017). On the other hand, antiadhesive barrier layers effectively reduced the incidence of adhesion between the viscera and the implanted mesh, one of the main cause of patient discomfort (Dinsmore et al., 2000).

Electrospinning (ES) is a versatile and simple technique for obtaining non-woven nanofibrous membranes with high surface-to-volume ratio, porosity, pore interconnectivity, and other easy-tailorable properties (Greiner and Wendorff, 2007). Electrospun membranes are widely employed as scaffolds in tissue engineering applications since their ECM-like three-dimensional architecture is able to support cellular adhesion, spreading and functions, while the intrinsic porosity and pore interconnectivity facilitates angiogenesis, ultimately promoting tissue homeostasis and repair (Agarwal et al., 2008; Wang et al., 2013). Nonetheless, few literature studies adopted ES as coating method to increase mesh biocompatibility (Plencner et al., 2015; East et al., 2018; Liu et al., 2019), although some nanofiber-based meshes were developed (Ebersole et al., 2012; Chakroff et al., 2015; Jangö et al., 2017).

One main advantage is that nanofibers can be obtained from many natural and synthetic polymers, copolymers, blends or composites (Greiner and Wendorff, 2007). Some of the most employed polymers include gelatin (Gel) (Tonda-Turo et al., 2013; Aldana and Abraham, 2017), a water-soluble protein derived from the partial denaturation of collagen, extremely biocompatible, with low antigenicity, and many integrin-binding sites, and polycaprolactone (PCL) (Azimi et al., 2014), a synthetic hydrophobic polyester, with a semi-crystalline structure, and slow degradation rate. PCL biocompatibility has been extensively studied, and many PCL-based medical devices have the US Food and Drug Administration's approval to be used in humans. Some of these include absorbable sutures (e.g., Monocryl™ and Maxon™), root canal filling materials (Resilon™), patches for tendon (Artlemon®), sheaths for peripheral nerve regeneration (Neurolac®), 3D printed bone implants (Osteopore™), or drug delivery systems (Capronor®).

Many research groups have already developed nanofibrous membranes based on PCL and Gel blends, allowing to obtain biomimetic membranes with suitable physico-chemical and mechanical properties, and improved biological behavior. Usually highly toxic solvents, such as hexafluoro-2-propanol (HFIP) (Kuppan et al., 2013) and trifluoroethanol (Alvarez-Perez et al., 2010), were used to fabricate PCL/Gel mats, but researchers recently aimed to find alternative and less toxic solutions. Nanofibers were electrospun from a solution of PCL, dissolved in chloroform/methanol, and Gel, dissolved in acetic acid (AA)/water (Gautam et al., 2013). In another work (Binulal et al., 2014), composite PCL and Gel nanofibers were also obtained from a diluted AA/ethyl acetate mixture. Alternatively, AA and formic acid (FA) have been proposed as very promising aqueous-based solvents for both polymers. Denis et al. compared composite PCL and Gel nanofibers electrospun using either HFIP or acetic and formic acids (9:1 ratio) solvent mixture, concluding that, in the latter case, nanofibers with similar characteristics could be obtained (Denis et al., 2015). Similarly, Gil-Castel and co-workers used the same solvent system with a different ratio (1:1 ratio), investigating the effects of different dissolution times on nanofibers physico-chemical properties (Gil-Castell et al., 2018).

The aim of this work is to preliminary assess the potential of combining a nanostructured electrospun membrane based on PCL/Gel blend and a commercial PP mesh in order to develop a novel multicomponent hernia mesh device (meshPCL-Gel). Compared to other strategies that mainly relies on the modification of mesh filaments surface, electrospun membranes deposited on the mesh have the advantage of operating at different scale lengths: (i) at the nanoscale, to resemble the complex three-dimensional structure and composition of the native ECM microenvironment; (ii) at the micro- and macroscale, to cover the whole mesh surface, including the empty pore spaces, where cells lack of a substrate for adhesion.

Therefore, upon implantation, the nanostructured coating could further promote and accelerate the integration of the mesh at the abdominal site. As a preliminary validation, the physico-chemical and mechanical properties of the PCL-Gel nanofibrous membrane were evaluated and compared with those of singular PCL and Gel membranes. Then, the *in vitro* response of human fibroblasts to either PCL-Gel membranes or the meshPCL-Gel device was assessed. The obtained results evidenced the good cell viability and non-toxicity of the multicomponent meshPCL-Gel device and its potentiality as novel solution for abdominal wall hernia repair.

## Materials and Methods

### Materials

Evolution Blue PP mesh (EV3PB) samples were kindly provided by Dipro Medical Devices S.r.l. Polycaprolactone (PCL, Mn 70,000–90,000 g/mol), gelatin type A from porcine skin (Gel), formic acid (FA, ACS reagent ≥96%), acetic acid (AA, ≥99,7%), (3-Glycidyloxypropyl)trimethoxysilane (GPTMS, ≥98%), and phosphate buffer saline (PBS) tablets were purchased from Sigma-Aldrich (Milan). Chloroform (CHL, RPE, stabilized with ethanol), and ethanol (EtOH) were purchased from Carlo Erba Reagents. Collagenase type I (>125 units/mg) was purchased from Life Technologies Italia. All solvents were of analytical grade and used without further purification.

### Nanofibrous Membranes Preparation

#### Solutions Preparation

PCL solution was prepared by dissolving 12% wt/v PCL in CHL/FA (70/30 v/v). First, PCL was dissolved in CHL, then FA was added, and the solution was stirred for additional 30 min. For Gel solution, Gel was dissolved in AA/milliQ water (20/80 v/v) at 20% wt/v until complete dissolution, then 92 μL/g of GPTMS were added and the solution was stirred for additional 40 min before the electrospinning process. The PCL and Gel blend (PCL-Gel) solution was prepared in an AA/FA (1:1 v/v) solvent system, with 15% wt/v total solid concentration (80:20 PCL:Gel wt/wt). Briefly, PCL and Gel were dissolved together in the solvent system for 24 h, then 3.68% v/v of GPTMS was added, and the solution was stirred for additional 40 min before the electrospinning process. All the solutions were prepared at room temperature.

#### Electrospinning Process

Nanofibrous membranes were prepared using a Linari Engineering S.r.l electrospinning equipment. Solutions were loaded in 5 mL luer-lock glass syringes with a 21-gauge metallic needle, and electrospun at room temperature onto a plane collector. Table 1 summarizes the process parameters used to obtain, PCL, Gel, and PCL-Gel nanofibers.

**Table 1:** Electrospinning process parameters.

	**Voltage (kV)**	**Flow rate (mL/h)**	**Distance (cm)**
PCL	20	1.5	20
Gel	28	1.2	13
PCL-Gel	21	0.5	13

### Preparation of the Multicomponent Hernia Mesh (meshPCL-Gel)

EV3PB meshes were first precoated by dipping them in a 1% wt/v PCL solution in CHL for 10 min. After the complete solvent evaporation at room temperature, precoated meshes were attached to the collector with a conductive tape for the deposition of PCL-Gel nanofibers. Supplementary Figure 1 reports the scheme of the fabrication steps.

### Characterization

#### Morphological Analysis

Morphological evaluations of PCL, Gel, PCL-Gel, and meshPCL-Gel were conducted by scanning electron microscopy (SEM, LEO – 1430, Zeiss), or field emission scanning electron microscopy (FESEM, ZEISS Supra 40). Before analysis, samples were coated with a thin gold layer for SEM evaluations, or a thin chromium layer for FESEM evaluations. The average nanofibers diameters were calculated from SEM images (*n* > 100 measurements per sample type) *via* ImageJ software.

#### Attenuated Total Reflectance Fourier Transform Infrared (ATR-FTIR) Spectroscopy

ATR-FTIR spectroscopy was performed on PCL, Gel, and PCL-Gel, employing a Spectrum 100 instrument (Perkin Elmer) equipped with a diamond crystal. Spectra were obtained at room temperature in the 4,000 and 600 cm^−1^ wavenumber range, at a resolution of 4 cm^−1^ and averaged over 32 scans. Spectra were analyzed by Spectrum software.

#### Differential Scanning Calorimetry (DSC)

Thermal analyses (Mettler Toledo) of PCL, Gel, and PCL-Gel were performed through differential scanning calorimetry (DSC) on samples (5–10 mg) packed in aluminum pans. Three scans were performed between T_amb_ and 200°C at a heating rate of 10°C min^−1^ with 5 min isotherm at each target temperature, under nitrogen atmosphere.

#### Water Contact Angle (WCA)

Membranes wettability was assessed with Krüss Drop Shape Analyzer. The static WCA was obtained at *t* = 0 *via* the sessile droplet method and analyzed through Krüss Drop Shape Analysis software. A 2 μL drop of milliQ water was deposited on the surface, then the initial WCA was calculated with an ellipse fitting method (*n* = 6 per membrane type).

#### Hydrolytic Degradation Test

Hydrolytic degradation tests on PCL-Gel and meshPCL-Gel samples were carried out in PBS at 37°C for 1, 3, 7, 14, 21, and 28 days; PBS solution was replaced every 3 days. After each time step, samples were removed from PBS, rinsed twice with milliQ water, and freeze-dried for 24 h (Scanvac CoolSafe). The weights of PCL-Gel (*n* = 4 per time step, 5–12 mg), and meshPCL-Gel (*n* > 3 per time step, 2 × 1 cm^2^) samples were recorded before (*W*_0_) and after each time step (*W*_*f*_*)*. The percentage of remaining weight *W*_*R%*_ was calculated using the equation:

(1) $$\begin{array}{r} W_{R\%}\;=\;\left(W_{f}/W_{0}\right)\cdot \mathrm{100} \end{array}$$

Morphological features of degraded PCL-Gel and meshPCL-Gel samples were analyzed through SEM or FESEM analysis at different magnifications.

#### Enzymatic Degradation Test

Enzymatic degradation tests on PCL-Gel and meshPCL-Gel samples were carried out at 37°C for 1, 3, 7, 14, 21, and 28 days. Samples were immersed in 3 mL of PBS containing 0.2 mg/mL of collagenase (Eskandarinia et al., 2020). Enzymatic solutions were replaced every 3 days. After each time step, weight loss and morphology of degraded PCL-Gel (*n* = 4 per time step, 2 × 2 cm^2^) and meshPCL-Gel (*n* > 3 per time step, 2 × 1 cm^2^) samples were evaluated following the protocols described in paragraph Hydrolytic Degradation Test.

#### Uniaxial Tensile Test

Mechanical properties of the three types of nanofibrous membranes, along with those of EV3PB and meshPCL-Gel were obtained through a tensile test (MTS QTest/10) machine. Dog-bone shaped samples (5 mm width, 13 cm gauge length) were tested at 2 mm/min until rupture using a 10N load cell. Thickness was measured at the center of each sample with a micrometer. From the stress-strain curves, the Young's modulus (*E*), ultimate tensile stress (σ_*R*_), and strain at rupture (ε_*R*_) were calculated (*n* ≥ 3).

### Biological Evaluation

#### *In vitro* Studies

For the biological validation, meshPCL-Gel and PCL-Gel samples were placed in multiwells (12 wells, 2.1 cm diameter). meshPCL-Gel samples were hand-cut in circles of 2.1 cm to fit wells dimensions, whereas PCL-Gel nanofibers were electrospun onto circular cover glasses (2 cm diameter). Before the evaluations, all samples were sterilized for 1 h under UV in a sterile vertical laminar air flow cabinet and maintained in a sterile way until use.

#### Viability Assay

A test of direct cytotoxicity following ISO 10993-5:2018 was carried out using human fibroblast cell line BJ (ATCC, Rockville, MD, USA). Cells were routinely cultured in high-glucose DMEM (Dulbecco's Modified Eagle Medium) supplemented with 2 mM glutamine, 1% antibiotic/anti-mycotic solution and 10% fetal bovine serum (FBS, v/v). For the evaluation, cells were seeded on sterilized samples at the concentration 10,000/cm^2^ and maintained in an atmosphere of 5% CO_2_ and 95% air at 37°C. Cells grown on multiwells in absence of any sample were used as control. Each type of samples and controls were tested in triplicate.

At the different experimental times (24 h, 72 h, 7 days), cell growth was determined by MTT assay directly on attached cells. Briefly, cells grown on meshPCL-Gel and PCL-Gel and control cells were added with 30 μL of 4.5-dimethylthiazol-2-yl-,5-diphenyltetrazolium bromide (MTT, 5 mg/mL) in PBS solution, and incubated for 3 h at 37°C in a humidified atmosphere of 5% CO_2_ in air. After, the culture medium was removed and 150 μL of dimethyl sulfoxide (DMSO) were added to each well. After 20 min of incubation at 37°C, the absorbance was measured at 595 nm using a spectrophotometer DU-800 (Beckman Coulter). The absorbance of each sample was expressed as absolute absorbance values or as percentage of the absorbance of the control cells at the corresponding experimental time.

For morphological observations by FESEM analysis, 48 h after cell seeding, meshPCL-Gel and PCL-Gel samples were removed from the multiwell, washed twice with PBS solution and fixed with 2.5% glutaraldehyde in 0.1 M phosphate buffer, pH 7.4, for 30 min at 4°C. Samples were then dehydrated with slow water replacement by a series of graded ethanol (EtOH) solutions (immersion in 30–50–70–80–90–95% v/v of EtOH, 15 min each) with final dehydration in absolute EtOH (100% v/v, 15 min) before critical-point drying.

#### Cytotoxicity Assay

The possible cytotoxic effect due to the growth of cells on different types of samples was investigated by evaluating the release of lactate dehydrogenase (LDH) in the culture medium. The analysis was carried out at three experimental times: 24, 48, and 72 h after cell seeding. The LDH activity was determined evaluating at 340 nm the consumption of NADH using a spectrophotometer DU-800 (Beckman Coulter). The NADH consumption directly correlates with the activity of the enzyme that was expressed as nmoles of NADH consumed/min/mL of culture medium. Each type of samples and controls were tested in triplicate. On the same culture medium, the number of detached cells was evaluated using a Burker chamber.

### Statistical Analyses

Experimental data are expressed as mean ± standard deviation. Differences between group means were assessed by using Prism GraphPad software (v8.2.0). Data were analyzed by unpaired two-tailed Student's *t*-test or one-way Anova analysis of variance with a 95% confidence interval, followed by Bonferroni *post-hoc* test corrected for multiple comparisons (significances set at α = 0.05).

## Results

### Nanofibrous Membranes Characterization

#### Scanning Electron Microscopy

**Figure 1:**
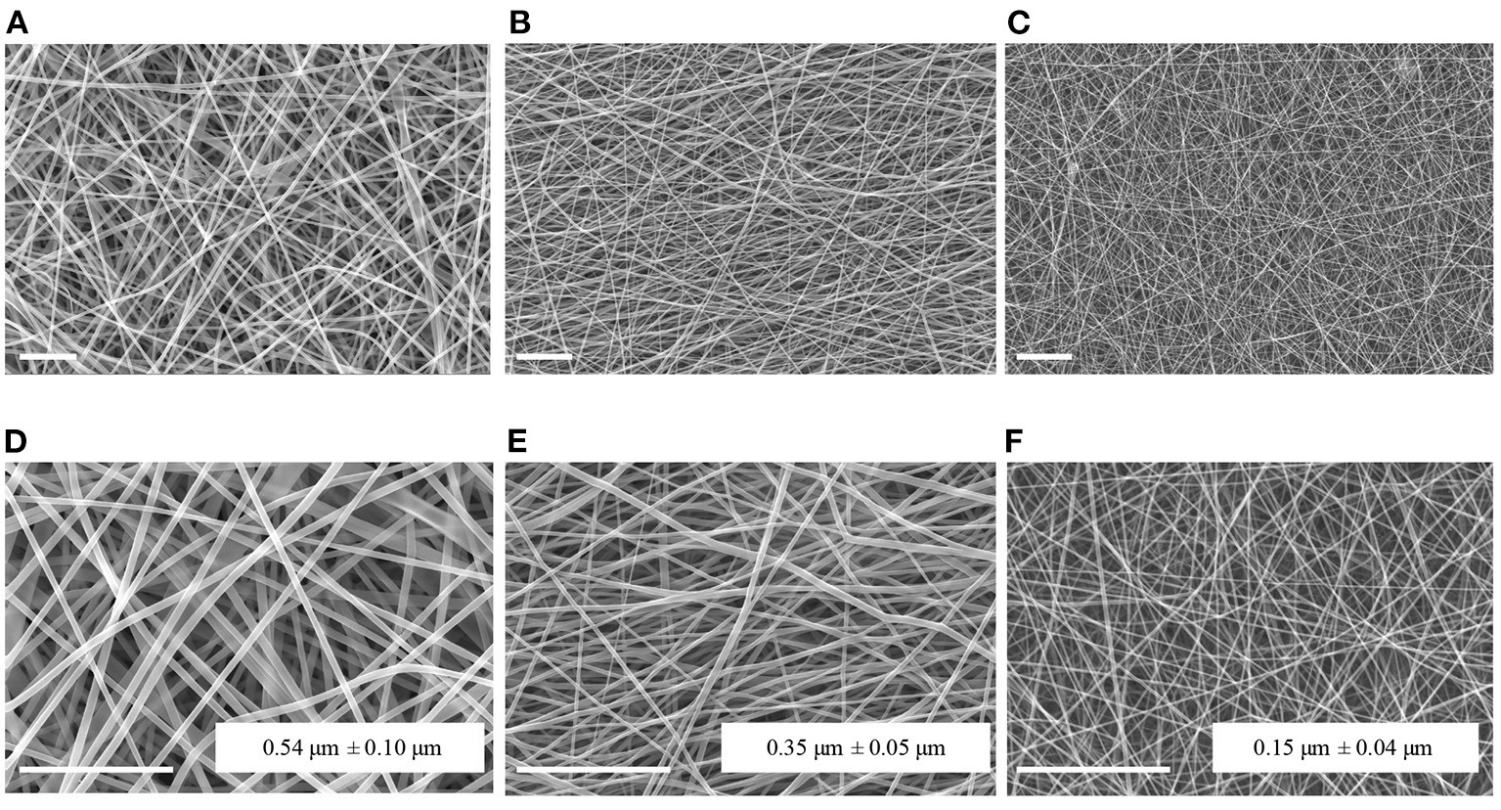
SEM micrographs and average fiber diameters of PCL **(A,D)**, Gel **(B,E)**, and PCL-Gel **(C,F)** nanofibers at different magnifications (**A–C**: 2kx; **D–F**: 5kx). Scale bars equal to 10 μm.

Figure 1 reports the SEM images of PCL, Gel, and PCL-Gel electrospun membranes at two different magnifications. Bead-free and highly porous nanofibrous membranes were effectively obtained by applying the electrospinning parameters reported in Table 1 at room temperature. PCL nanofibers had the highest average diameter (0.54 ± 0.10 μm), while Gel displayed nanofibers with a average diameter of 0.35 ± 0.05 μm. Lastly, PCL-Gel blend mats showed ultrathin and homogeneous smooth nanofibers with an average diameter of 0.15 ± 0.04 μm.

#### ATR-FTIR

**Figure 2:**
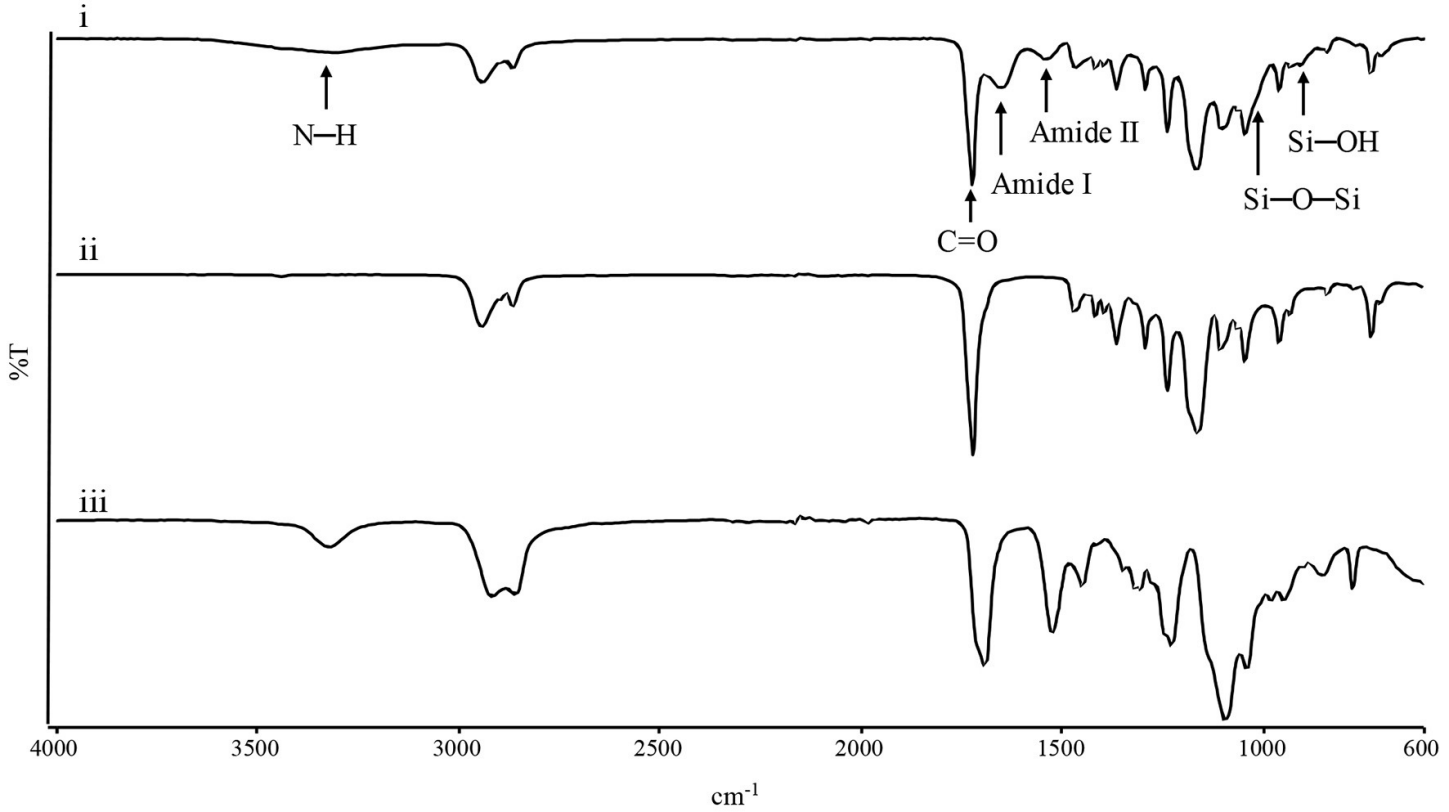
ATR-FTIR spectra of (i) PCL-Gel, (ii) PCL, and (iii) Gel membranes.

Infrared spectroscopy was performed to evaluate chemical composition of the electrospun mats. Figure 2 shows the spectra of the three membranes. In the PCL spectrum, typical adsorption bands could be detected at 2,943 cm^−1^ (asymmetric CH_2_ stretching), 2,865 cm^−1^ (symmetric CH_2_ stretching), 1,723 cm^−1^ (C=O vibrations), 1,293 cm^−1^ (CO and CC stretching), 1,250 cm^−1^ (asymmetric C-O-C stretching), and 1,185 cm^−1^ (symmetric C-O-C stretching) (Gautam et al., 2013). In the Gel spectrum, typical absorption bands were found at 3,420 cm^−1^ (N-H stretching), 1,692 cm^−1^ (amide I, C=O vibrations), 1,531 and 1,234 cm^−1^ (amide II and amide III, originating from C-N stretching and N-H in-plane bending), while the absorption bands at 1,104, 1,064, and 950 cm^−1^ referred to the Si–O–Si bonds and Si-OH stretching associated to the successful crosslinking between Gel and GPTMS (Tonda-Turo et al., 2013; Gnavi et al., 2015). PCL-Gel membranes showed the characteristic absorption bands of either PCL, Gel, and GPTMS, such as the sharp peak of the PCL carbonyl group at 1,726 cm^−1^ and the peaks attributable to Gel-GPTMS crosslinking, at 1,025 and 905 cm^−1^. However, the PCL-Gel spectrum shows a slight shift of the amide I and amide II absorption bands compared to those of Gel spectrum, respectively at 1,652 and 1,542 cm^−1^.

#### DSC

**Figure 3:**
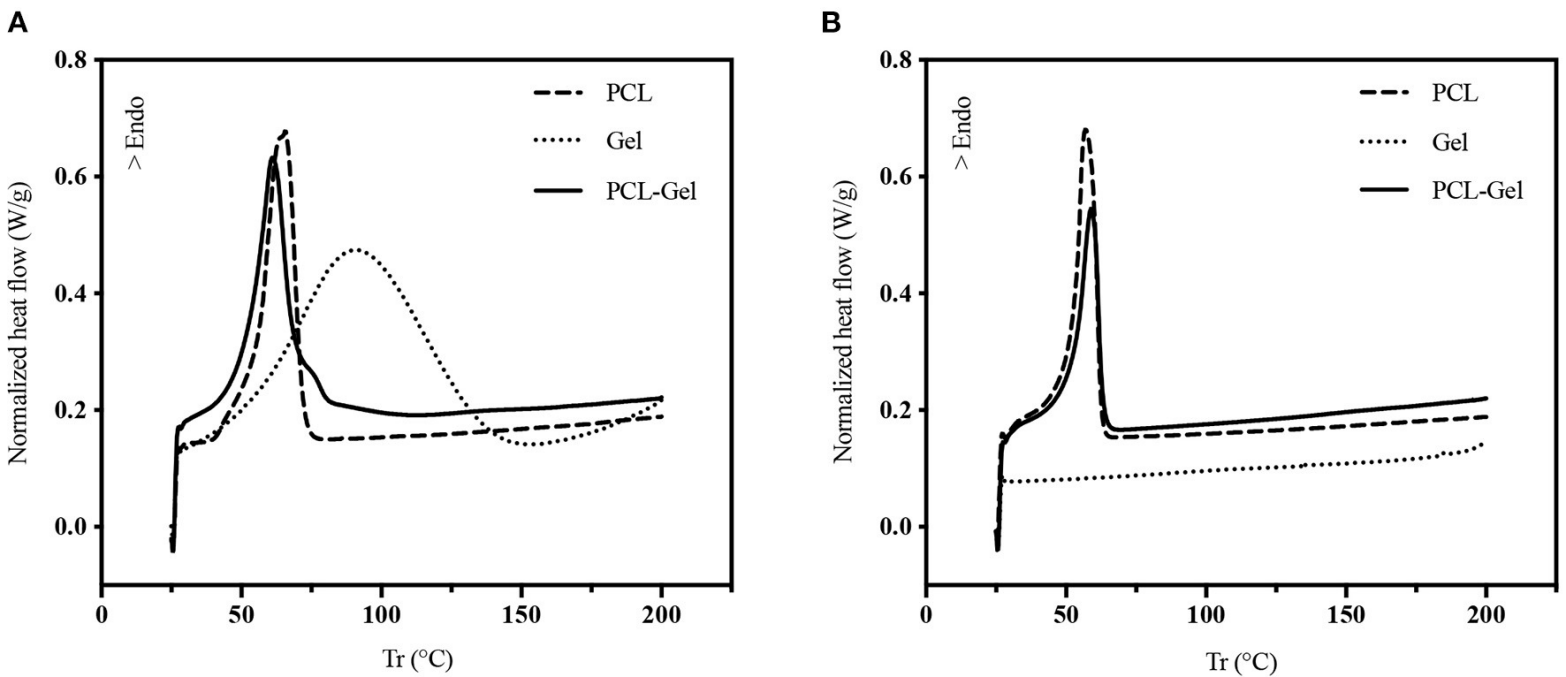
DSC thermogram of the **(A)** 1st and **(B)** 2nd heating cycle (10°C/min) of PCL, Gel, and PCL-Gel membranes.

DSC analysis was employed to study the thermal behavior of PCL-Gel nanofibers compared to PCL and Gel ones. Figure 3A shows the DSC thermograms of the first heating cycle. For PCL nanofibers, the endothermic peak relative to the polymer melting temperature (T_m_) was found at around 65.67°C, while GPTMS-crosslinked Gel nanofibers showed a broad endothermic peak between 40 and 150°C, with a peak temperature of 90.83°C, which was attributed to multiple overlapping thermal events, such as Gel denaturation and water evaporation (Zhang et al., 2006; Tonda-Turo et al., 2011; Vilches et al., 2019). In the PCL-Gel curve, the PCL-melting peak was shifted to a lower value, around 61.17°C, while a second small peak relative to the Gel phase was present around 80°C. Looking at thermograms of the second heating cycle (Figure 3B), the peaks related to the Gel-phase disappeared, due to either Gel denaturation and water evaporation, while the sharp peaks of the PCL-phase were still present. No significant differences are found between PCL and PCL-Gel curves.

#### Membranes Wettability

**Figure 4:**
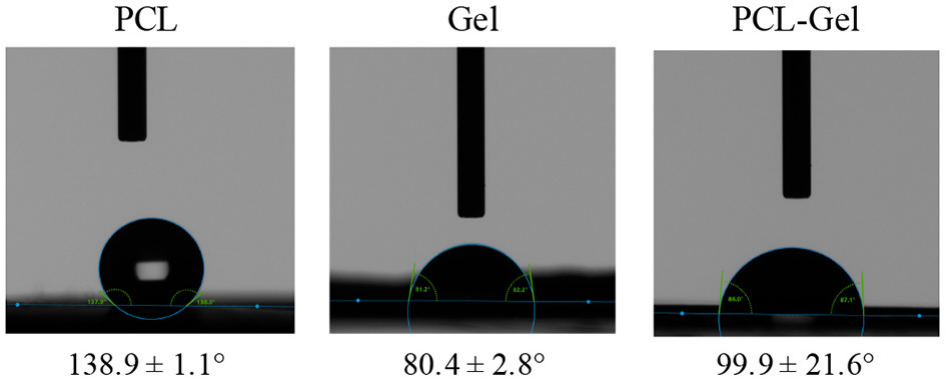
Optical images of the droplets at *t* = 0 for wettability measurements and average water contact angle of PCL, Gel, and PCL-Gel electrospun membranes.

Static water contact angle was performed to assess the wettability of the electrospun membranes. As shown in Figure 4, PCL membranes displayed a strong hydrophobic behavior (138.9 ± 1.1°) whereas Gel membranes had a water contact angle 80.4 ± 2.8°; PCL-Gel nanofibres were less hydrophobic (99.9 ± 21.6°) than the PCL ones, due to the presence of the amino and carboxyl functional groups (Ren et al., 2017). Nonetheless, the wide range of values obtained for PCL-Gel (from 69 to 124°) suggested that there were regions with different wettability on the sample surface.

#### Tensile Properties

**Figure 5:**
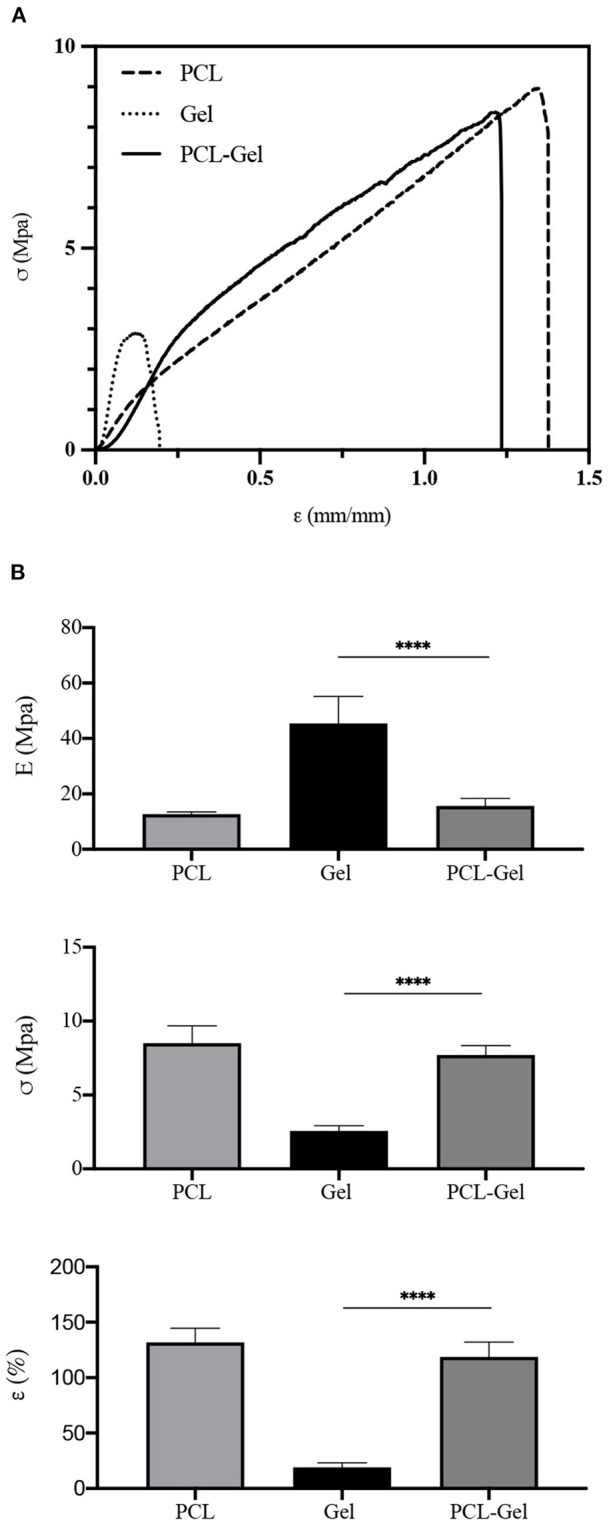
Mechanical properties obtained from uniaxial tensile test until rupture of PCL, Gel, and PCL-Gel electrospun membranes. **(A)** Typical stress-strain curves of each type of membrane, and **(B)** histograms of the average Young's modulus (**E**), ultimate tensile stress (**σ**_**R**_), and strain (**ε**_**R**_) at rupture. Data are presented as mean ± standard deviation (*n* > 3). Asterisks denote statistically significant differences (****p < 0.00001), as determined by one-way Anova analysis of variance followed by a *post-hoc* Bonferroni test.

Mechanical properties of PCL, Gel, and PCL-Gel membranes were obtained from uniaxial tensile tests until rupture. From the stress-strain curves (Figure 5A), the average Young's modulus (*E*), ultimate tensile stress (σ_*R*_), and strain at rupture (ε_*R*_) were calculated, and results are summarized in Table 2. As evident in the histograms reported in Figure 5B, Gel membranes showed the highest stiffness (45.5 ± 9.7 MPa) but the lowest ultimate tensile stress (2.6 ± 0.3 MPa) and elongation (19.1 ± 4.1%), being the most rigid and fragile membranes. On the contrary, the PCL ones were the most ductile, with the lowest stiffness (12.7 ± 0.8 MPa) and the highest ultimate tensile stress (8.5 ± 1.2 MPa) and elongation (131.8 ± 12.8%). Overall, PCL-Gel membranes properties were not significantly different from those of the PCL ones, however the presence of Gel seemed to slightly influence the mechanical behavior of blend membranes with an increase in stiffness (15.7 ± 2.7 MPa) and a decrease in strength and elongation (7.7 ± 0.6 MPa and 118.8 ± 13.2%, respectively).

**Table 2:** Average Young's modulus (**E**), ultimate tensile stress (**σ**_**R**_), and strain at rupture (**ε_R_**), calculated from ***n*** samples.

	***E*** **(MPa)**		***σ_R_* (MPa)**	***ε_R_* (%)**	***n***
PCL	12.7 ± 0.8		8.5 ± 1.2	131.8 ± 12.8	7
Gel	45.5 ± 9.7		2.6 ± 0.3	19.1 ± 4.1	3
PCL-Gel	15.7 ± 2.7		7.7 ± 0.6	118.8 ± 13.2	4
	***E**_***A***_* **(MPa)**	***E**_***B***_* **(MPa)**			
EV3PB	1.2 ± 0.1	3.1 ± 1	1.2 ± 0.7	41.4 ± 16.4	6
meshPCL-Gel	1.3 ± 0.4	3.0 ± 0.2	1.0 ± 0.7	41.4 ± 19.1	6

#### PCL-Gel Hydrolytic and Enzymatic Degradation

**Figure 6:**
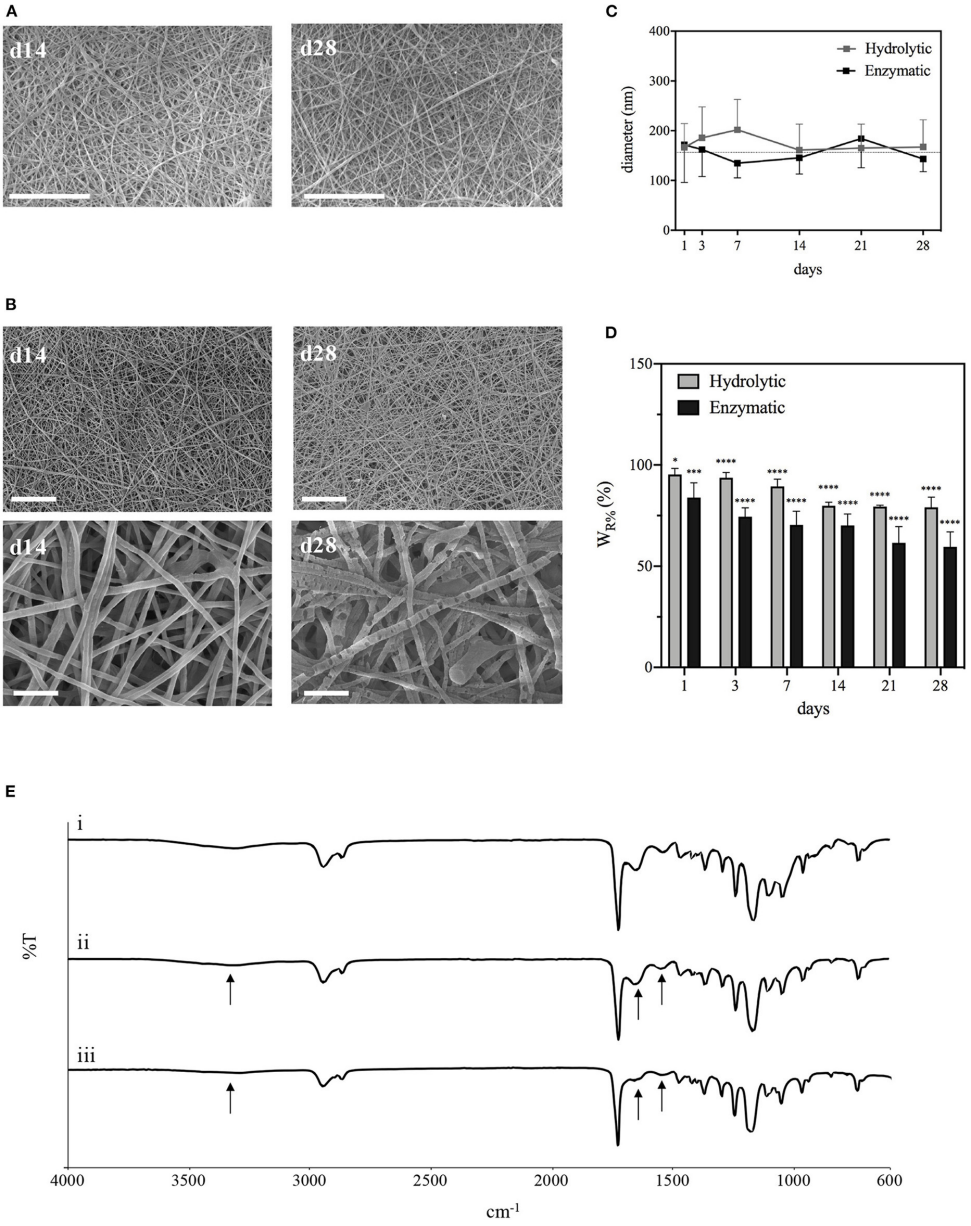
Characterizations of PCL-Gel membranes degraded in PBS or collagenase solutions at 37°C. **(A)** SEM images of PCL-Gel after 14 or 28 days of hydrolytic degradation at 5kx magnification; **(B)** FESEM images of PCL-Gel after 14 or 28 days of enzymatic degradation at different magnifications (left 5kx; right 50kx). Scale bars equal to 10 μm for 5kx and 1 μm for 50kx. **(C)** Variations in nanofibers diameter and **(D)** membranes weight loss; **(E)** comparison of FTIR spectra of PCL-Gel membranes at (i) day 0, and day 28 of (ii) hydrolytic and (iii) enzymatic degradation. Data are presented as mean ± standard deviation (*n* > 3). Asterisks denote statistically significant differences compared to *t* = 0 (*p < 0.01, ***p < 0.0001, and ****p < 0.00001), as determined by analysis of variance followed by a *post-hoc* Bonferroni test.

The degradation behavior of PCL-Gel membranes after incubation either in PBS or collagenase solutions up to 28 days was evaluated through SEM/FESEM analysis and the quantification of loss of weight. After 28 days of hydrolytic degradation, there were no significant changes in nanofibers morphology (Figure 6A). In the first 7 days, nanofibers swelled, and the average diameter increased until reached a maximum of 0.20 ± 0.06 μm at day 7; then it decreased again and remained almost steady between the 14th and 28th day (Figure 6C). Similarly, samples weight loss increased during the first days, reaching a plateau between the 14th and 28th day, with maximum weight loss at the end of the test estimated around 20% (Figure 6D). With regards to enzymatic degradation, PCL-Gel nanofibers exhibited greater weight loss than those in PBS solution. After 1 day, weight loss was about 16% and steadily increased up to 40% after 28 days (Figure 6D). However, nanofibers did not swell, fuse, or completely degrade upon collagenase exposure. The average diameters were overall lower than those measured during hydrolytic degradation and reached a minimum of 0.13 ± 0.3 nm at day 7 (Figure 6C). Although there were no visible signs of degradation in the overall appearance of PCL-Gel nanofibers both after 14 or 28 days, FESEM images at high magnification revealed that in the latter case nanofibers had undergone non-homogeneous degradation, which may be attributed to the selective degradation of the Gel phase within single nanofibers (Figure 6B). However, comparing the FTIR spectra of PCL-Gel samples before and after either hydrolytic or enzymatic degradation the absorption bands relative to Gel are still present even after 28 days, as indicated by the arrows in Figure 6E.

### meshPCL-Gel Device Characterizations

**Figure 7:**
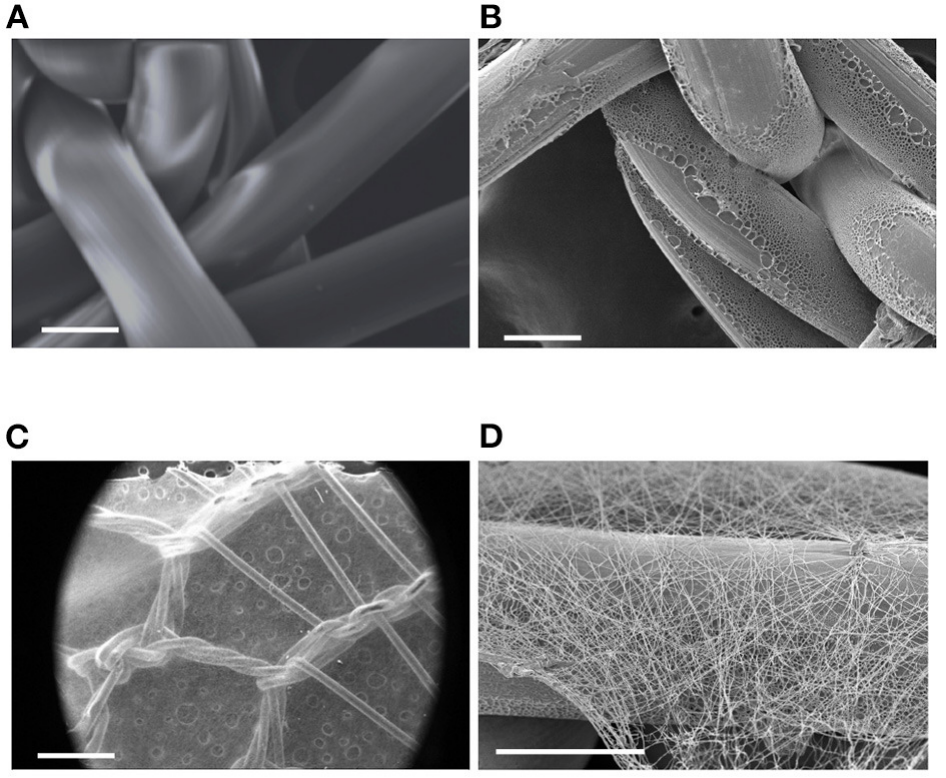
SEM images of: **(A)** pristine PP mesh, **(B)** PP mesh coated with PCL film, **(C,D)** meshPCL-Gel device at different magnifications. Scale bars equal to 100 μm **(A,B,D)**, and 1 mm **(C)**.

The meshPCL-Gel device was obtained by electrospinning PCL-Gel nanofibers onto PCL-precoated EV3PB mesh. A thin non-homogeneous layer of PCL was deposited on PP filaments (Figures 7A,B) to increase the adhesion between the mesh and the nanofibrous coating. PCL-Gel nanofibers adhered to the PCL-precoated mesh and formed a continuous layer on top of the mesh, with an average thickness of 15 ± 10 μm (Figures 7C,D). The time needed for covering a single piece of PCL-precoated PP mesh depended on mesh size. For instance, PCL-Gel nanofibers were electrospun on 7 × 2 cm^2^ mesh pieces for around 12 min. Qualitative tests on PCL-Gel membranes adhesiveness are presented as Supplementary Figure 2 and Supplementary Video 1.

#### Tensile Properties

**Figure 8:**
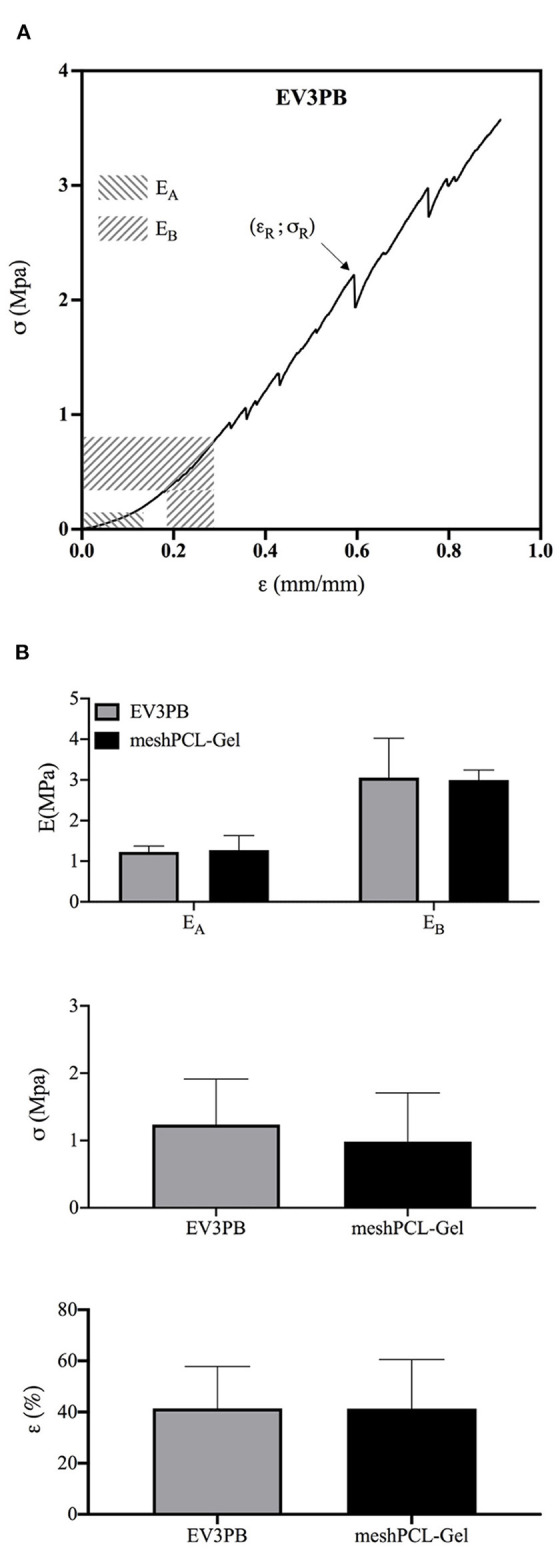
Mechanical properties obtained from uniaxial tensile test until rupture of EV3PB and meshPCL-Gel samples. **(A)** Typical stress-strain curve of each EV3PB meshes, and **(B)** histograms of the average Young's moduli (*E*_*A*_ and *E*_*B*_), ultimate tensile stress (**σ**_**R**_), and strain (**ε**_**R**_) at the first point of rupture, as indicated by the arrow. Data are presented as mean ± standard deviation (*n* = 6). Statistically analysis (Student's *t*-test) did not report significant differences between values.

Mechanical properties of the meshPCL-Gel device were evaluated and compare to those of the bare EV3PB mesh through uniaxial tensile test until rupture. EV3PB stress-strain curve (Figure 8A) displays bilinearity, having two different mechanical regimens: an initial low stiffness region, followed by a high stiffness region (Afonso et al., 2008; Jones et al., 2009). Therefore, two Young's moduli, namely *E*_*A*_ and *E*_*B*_, where calculated. For all specimens, the *E*_*A*_ region fell between 0 and 10% of deformation, whereas the *E*_*B*_ region fell between 15 and 30% of deformation. Maximum tensile stress (σ_*R*_) and maximum strain (ε_*R*_) were considered those at the first relevant point of rupture of meshes (as indicated by the arrow in Figure 8A). EV3PB stiffnesses, *E*_*A*_ and *E*_*B*_, were 1.2 ± 0.1 MPa and 3.1 ± 1 MPa, respectively; maximum stress and elongation at the first point of rupture were 1.2 ± 0.7 MPa and 41.4 ± 16.4%, respectively; meshPCL-Gel stiffnesses, *E*_*A*_ and *E*_*B*_, were 1.3 ± 0.4 MPa and 3.0 ± 0.2 MPa, respectively; maximum stress and elongation at the first point of rupture were 1.0 ± 0.7 MPa and 41.4 ± 19.1%, respectively. Statistical analysis (Student's *t*-test) revealed that the presence of PCL-Gel membrane did not influence the behavior of EV3PB under tension, with values comparable to the EV3PB's ones (Figure 8B and Table 2).

#### Hydrolytic and Enzymatic Degradation

**Figure 9:**
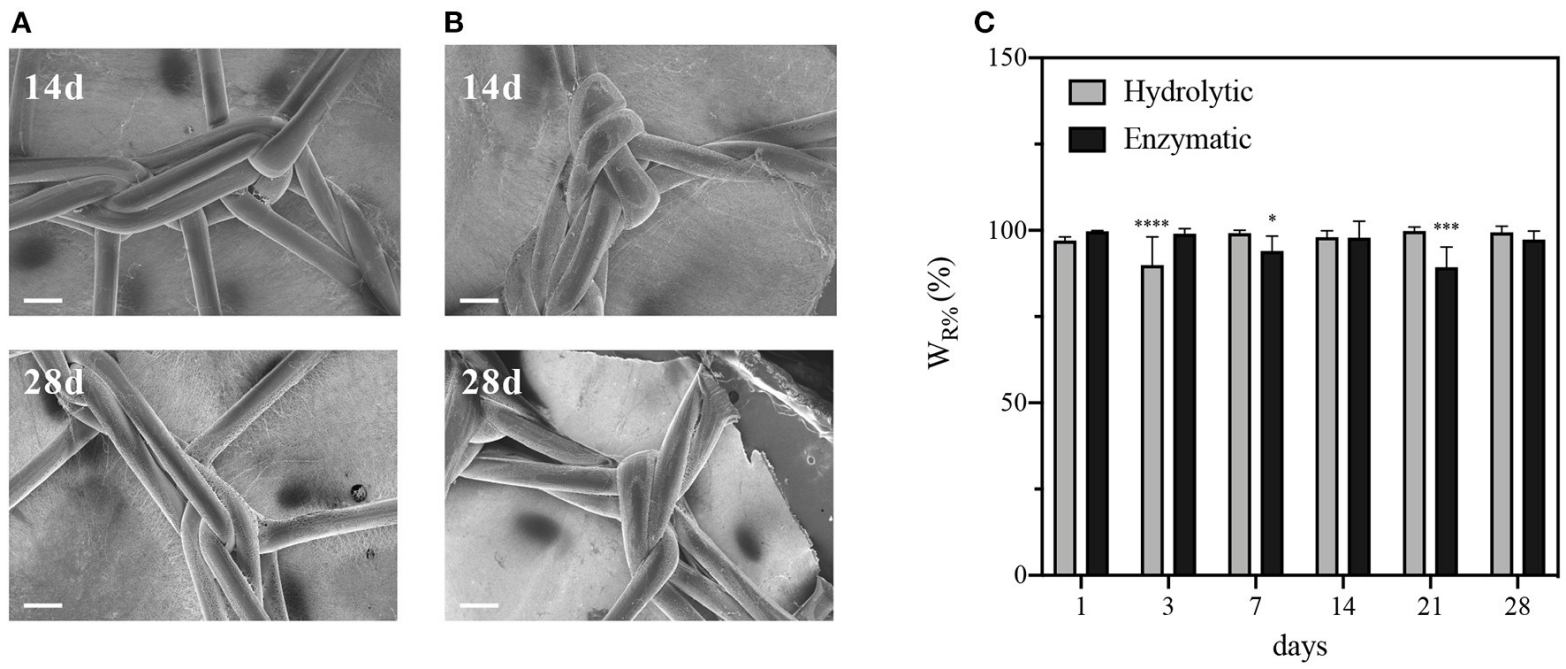
Characterizations of meshPCL-Gel degraded in PBS or collagenase solutions at 37°C. FESEM images after 14 or 28 days of **(A)** hydrolytic or **(B)** enzymatic degradation. **(C)** meshPCL-Gel weight loss. Data are presented as mean ± standard deviation (*n* > 3). Scale bars equal to 200 μm; Asterisks denote statistically significant differences compared to *t* = 0 (*p < 0.01, ***p < 0.0001, and ****p < 0.00001), as determined by analysis of variance followed by a *post-hoc* Bonferroni test.

Degradation of PCL-Gel membranes on the meshPCL-Gel device was carried out either in PBS or collagenase solutions, up to 28 days. The satisfactory appearance of the meshPCL-Gel device after 14 and 28 days was observed by FESEM, which also revealed the persistence of PCL-Gel nanofibers connecting with PP filaments (Figures 9A,B). The major contribution of meshPCL-Gel weight was given by EV3PB, which does not degrade under the condition used. Although weight losses are related solely to PCL-Gel degradation, these are referred to the initial weight of the entire device. Weight losses in both solutions ranged from 0.2 to 10% (Figure 9C). For samples in PBS solution, the percentage of remaining weight varied between a minimum of 89.9% and a maximum of 99.8%, being 99.4% after 28 days; similarly, it varied between a minimum of 89.3% and a maximum of 99.7%, being 97.3% after 28 days for samples in collagenase solution.

#### Biological Validation

**Figure 10:**
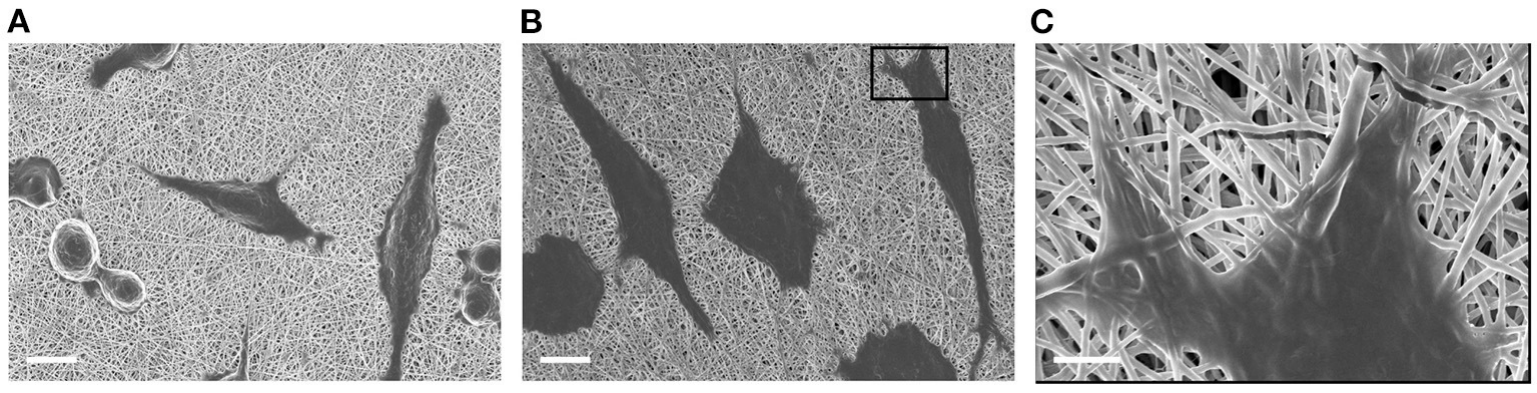
FESEM micrographs of BJ human fibroblasts grown on **(A)** PCL-Gel and **(B,C)** meshPCL-Gel substrates (PCL-Gel membrane side), after 48 h of culture. Scale bars equal to 100 μm **(A,B)** and 2 μm **(C)**.

The ability of human fibroblasts in colonizing both meshPCL-Gel device and PCL-Gel membranes was evidenced by optical (Supplementary Figure 3) and electron (Figure 10) microscopy. The latter also demonstrated that no morphological changes indicating cell suffering occur in cells grown on meshPCL-Gel and PCL-Gel, and that cells are able to proliferate, as evidenced by the mitotic event in Figure 10A.

**Figure 11:**
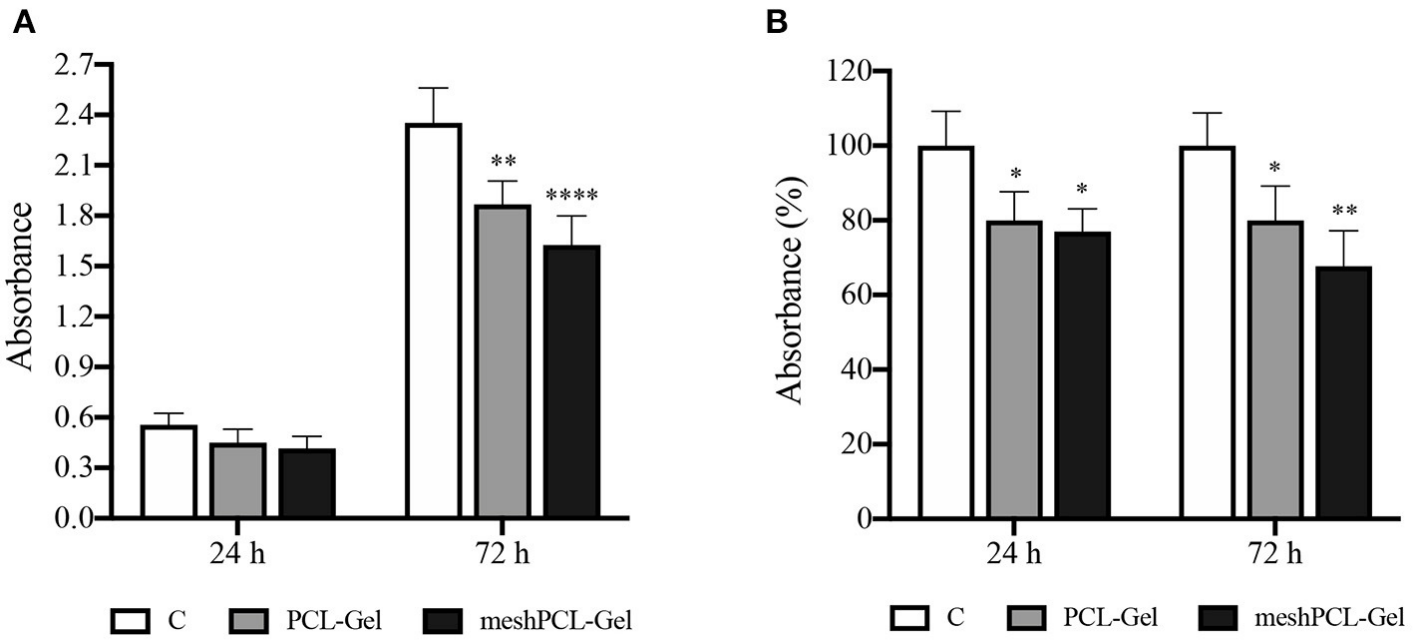
Growth of BJ human fibroblasts seeded onto the substrates tested. Values are expressed as **(A)** absolute absorbance at 595 nm, and **(B)** as percentage of control values. Data are presented as mean ± standard deviation (*n* = 3). Asterisks denote statistically significant differences compared to *t* = 0 (*p < 0.01, **p < 0.001, and ****p < 0.00001), as determined by analysis of variance followed by a *post-hoc* Bonferroni test.

MTT measures the mitochondrial metabolic activity, therefore is an indicator of cell viability. Results from MTT test demonstrated that the number of cells grown on meshPCL-Gel and PCL-Gel increased during the experimental times, as evidenced by the increase of absolute absorbance values, even if the proliferation is lower in comparison with control cells until 72 h (Figure 11). At both experimental times, the reduction of growth was of about 80 and 70% for PCL-Gel and meshPCL-Gel, respectively. Preliminary not reported results at 7 days after cell seeding seem to indicate that cells seeded on meshPCL-Gel and PCL-Gel restart to grow.

**Figure 12:**
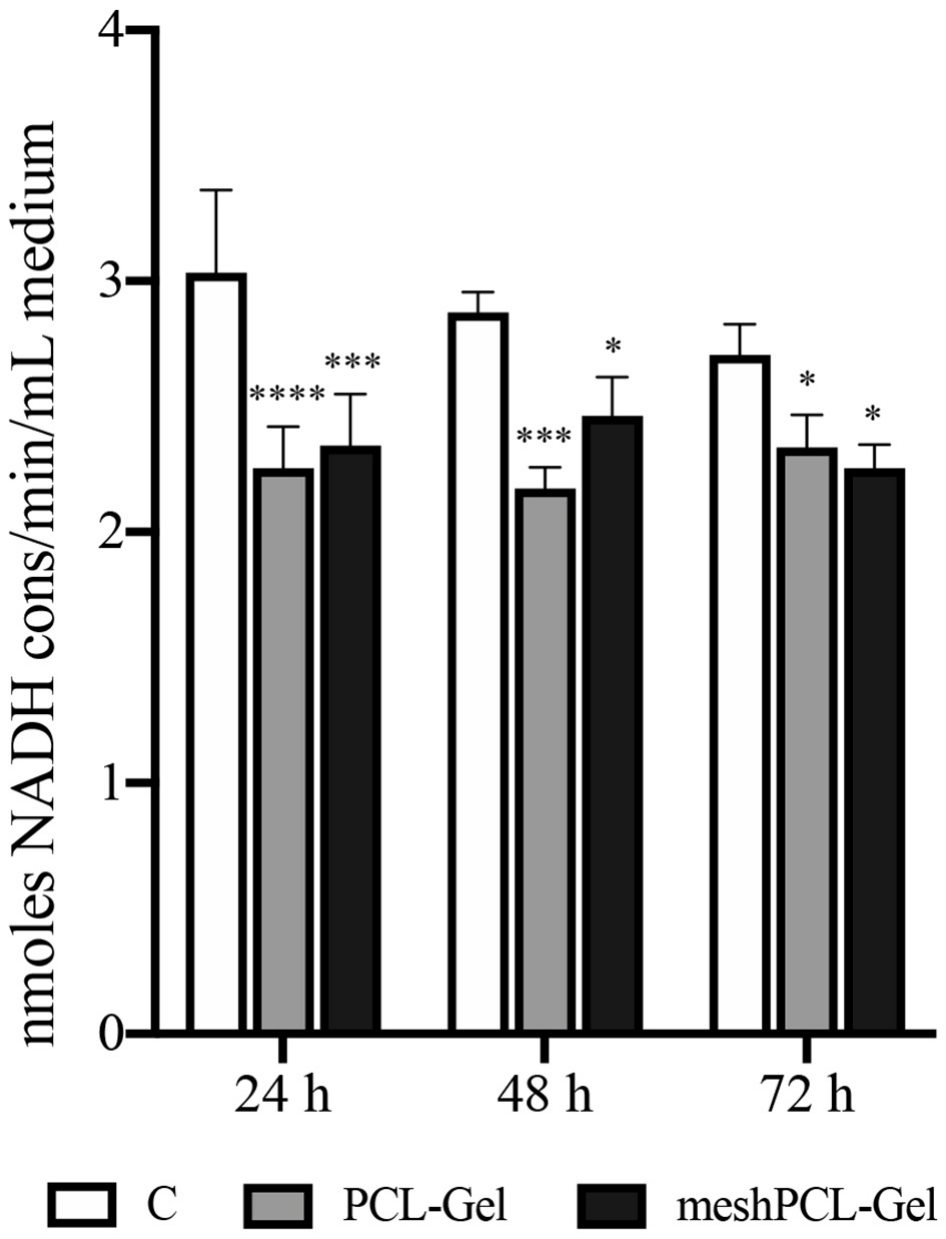
Lactate dehydrogenase release in the culture medium. Values are expressed as nmoles of NADH consumed per min and per mL of culture medium. Data are presented as mean ± standard deviation (*n* = 3). Asterisks denote statistically significant differences compared to *t* = 0 (*p < 0.01, ***p < 0.0001, and ****p < 0.00001), as determined by analysis of variance followed by a *post-hoc* Bonferroni test.

Then, the LDH release in the culture medium was evaluated as an indicator of necrotic death. The LDH values obtained from cells grown onto all the substrates tested were not higher than those of the controls at each time step (Figure 12), this indicating no induction of cytotoxicity. Cell counting performed with a Burker chambers evidenced that no detached cells were present in the culture medium of all samples (data not reported).

## Discussion

For patients suffering from hernia conditions, implantation of synthetic meshes is the gold standard in repair surgery. Nonetheless, at 5 years from the first intervention, around the 5% of patients requires a second surgery for hernia recurrence or even mesh removal (Kokotovic et al., 2016). The complications that lead to such need are caused by multiple factors, either patient behavior (Burger et al., 2004), surgeons expertise (Lange et al., 2016), or prosthesis characteristics (Kayaoglu et al., 2005). Among them, the occurrence of a prolonged state of inflammation seems to be correlated with a higher recurrence incidence due to an extensive FBR. The inflammatory reaction of tissues to the implanted mesh strongly impacts the healing process. Mesh-induced FBR hinders tissue remodeling, hence mesh integration, leading instead to excessive fibrosis, wound contraction, fistulas, adhesion, and general post-operative pain (Klinge et al., 2002; Junge et al., 2012; Klinge and Klosterhalfen, 2012; Deeken and Lake, 2017).

The present study aimed at assessing the possibility to develop a novel multicomponent hernia mesh device by coating commercial PP meshes with a nanostructured electrospun PCL-Gel membrane. This bioactive membrane could help the process of mesh integration and tissue remodeling by providing a more cell-friendly environment which would mitigate the chronic FBR.

First, PCL and Gel defect-free nanofibrous membranes were successfully electrospun at room temperature (Figures 1A,B,D,E). The protocol for PCL allowed to obtain nanofibers with an average diameter of 0.54 ± 0.10 μm. Notably, Gel nanofibers with average diameter of 0.35 ± 0.05 μm were obtained at room temperature thanks to the addition of a small percentage of AA in the solvent system, which made the electrospinning of nanofibers feasible by hindering Gel gelation process (Erencia et al., 2015). GPTMS was also introduced as crosslinker for Gel to prevent its rapid dissolution in aqueous environments. Next, a new protocol for the production of PCL-Gel nanofibres was developed by dissolving PCL and Gel in an AA/FA solvent system and adding GPTMS as crosslinker for Gel. After 24 h, as required for polymers dissolution, no phase separation was observed, and the solution appeared transparent. Recently, FA/AA have been successfully used as an alternative and less toxic solvent system for PCL/Gel blends in place to the commonly used fluorinated alcohols (Denis et al., 2015; Gil-Castell et al., 2018). This hydrolytic system has been shown to promote the formation of hydrogen bonds between PCL and Gel thanks to the partial hydrolysis of both polymers, overcoming the poor miscibility typical of natural-synthetic polymers blends (Feng et al., 2012; Ren et al., 2017). As a matter of fact, in PCL-Gel FTIR spectra (Figure 2), the small shifts of the original absorption bands could be explained by the formation of hydrogen bonds (Chakrapani et al., 2012; Gil-Castell et al., 2020) or by the generation of intermolecular electrostatic interactions between the ester groups of PCL and the amine groups of Gel (Gautam et al., 2013). Similarly, with regards to thermal analysis (Figure 3), the small shift of PCL melting temperature in PCL-Gel DSC thermograms to a lower temperature could be related to the hydrolytic degradation affecting PCL polymer chains during solution preparation (Gil-Castell et al., 2017), but it could also reveal the entanglements between PCL and Gel polymer chains (Gil-Castell et al., 2018).

Ultrathin and smooth PCL-Gel nanofibers with an average diameter of 150 ± 40 nm were obtained by electrospinning (Figures 1C,F). Such small diameter was obtained thanks to presence of Gel, known to increase solutions conductivity—one of the parameters influencing nanofibers diameter (Son et al., 2004)—by introducing many ionizable groups (i.e., amino and carboxyl); this effect was further accentuated by the strong polar nature of FA (Van der Schueren et al., 2011; Denis et al., 2015). Nanofibers diameter is known to determine cell behavior and lower values have been shown to better replicate the architecture of the native ECM components (Hodgkinson et al., 2014).

Despite the electrospinning process appeared very stable and nanofibers displayed a uniform external morphology, the presence of some non-homogeneity within the core was hypothesized by others, for instance from bright field images of nanofibers presenting either bright and dark areas (Zhang et al., 2005; Denis et al., 2015). This hypothesis could be confirmed by the results obtained from contact angle measurement, which showed that PCL-Gel membranes had a wide range of wettability values (from 69 to 124°) that could derive from the presence of such regions having slightly variable composition. Nonetheless, the overall behavior of the PCL-Gel changed from highly hydrophobic to moderately hydrophilic thanks to Gel functional groups, and reached values more friendly to living cells (van Wachem et al., 1985; Binulal et al., 2014; Jiang et al., 2017; Ren et al., 2017). With regards to PCL-Gel mechanical properties (Figure 5), tensile tests results showed that their average stiffness, and maximum stress and elongation were around 16 MPa, 8 MPa, and 119%, respectively. Thus, the overall behavior of PCL-Gel was comparable to that of PCL, due to its higher content within the blend (80:20 PCL:Gel wt/wt). However, the presence of Gel improved PCL-Gel stiffness (+19%), may be due to the presence of physical interactions and entanglement between PCL and Gel chains, also in accordance with DSC analysis, and only modestly diminished membranes strength and lengthening ability (< −10% in both cases).

PCL-Gel hydrolytic or enzymatic degradation was tested over a period of 28 days. Membrane resistance in physiological conditions is a crucial parameter for cellular infiltration and, consequently, integration with the host tissue. Since PCL has a much slower degradation rate that Gel, which fast dissolves in aqueous environments without further crosslinking, the mass loss could be expected to be entirely ascribed to Gel, even supposing the GPTMS crosslinking would prevent this phenomenon. The gradual degradation of Gel in PCL-Gel membranes is considered beneficial in creating additional space for cells infiltration. Results from hydrolytic degradation showed that over the whole period nanofibers maintained their structural integrity almost unaltered (Figure 6A). Weight loss, greater during the first half of the test because of faster surface erosion, was only 20% of the initial weight at the end of the test. Literature reports controversial results on the degradation behavior of PCL-Gel-based membranes by hydrolysis. For instance, Kuppan et al. found that PCL-Gel membranes prepared in HFIP lost around the 70% of their initial weight after only 10 days (Kuppan et al., 2013), whereas Dulnik et al. account for a weight loss after 90 days <10% for samples prepared with HFIP, and 15% when prepared in AA/FA (Dulnik et al., 2016).

Enzymatic degradation of PCL-Gel was also evaluated as a more physiological scenario. Enzymes of the matrix metalloproteinases (MMPs) family are commonly present during wound healing and contribute to tissue remodeling (Nwomeh et al., 1998). Among MMPs, collagenases are responsible for breaking down the peptide bonds of collagen, however, they are also used to evaluate the enzymatic degradation of Gel (Kishan et al., 2015; Yang et al., 2018; Eskandarinia et al., 2020). During enzymatic degradation, PCL-Gel weight losses were greater than those induced by hydrolysis due to the selectivity of the collagenase enzyme. Differently from PBS, weight loss did not reach a plateau but steadily increased during the whole period, reaching a maximum of 40% after 28 days. Other research groups report faster rates for PCL/Gel electrospun scaffolds degraded in collagenase solutions, with weight losses around 70–80% after just 14 days (Chen et al., 2019; Eskandarinia et al., 2020). This discrepancy of results can be explained with the crosslinking strategies that have or have not been adopted. A general observation is that weight losses by collagenase exposure are greater than the actual percentage of Gel within the scaffolds. In our case, the value is twice the initial percentage of Gel within the PCL-Gel blend. These can be attributed to the emergence of defects along nanofibers due to Gel degradation, as confirmed by FESEM (Figure 6B). Defects accelerate and aggravate degradation by creating discontinuities that eventually lead to nanofibers fracture. However, in both degradation experiments, FTIR spectra revealed that Gel absorption bands were still visible on the 28th day, as a consequence of both the blending with PCL and the crosslinking with GPTMS.

Thereupon, these results evidence that the PCL-Gel nanofibrous membrane presents high stability, appropriate hydrophilicity (Gungor-Ozkerim et al., 2014; Ren et al., 2017), bioactivity, and superior mechanical properties when compared to PCL and Gel membranes alone. Accordingly, PCL-Gel were further used as nanostructured coating of EV3PB commercial PP hernia mesh.

The multicomponent meshPCL-Gel device is obtained with few and simple steps by covering EV3PB with the PCL-Gel nanostructured coating, which had an average thickness of few microns. The membrane successfully covered the empty areas of mesh pores and adhered to the underlying mesh, even in wet conditions and upon gentle rubbing or bending.

Simple uniaxial tensile tests were conducted on both EV3PB and meshPCL-Gel to assess whether the presence of the PCL-Gel membrane could influence EV3PB behavior. Two different mechanical regimens were found and two Young's moduli were calculated (Afonso et al., 2008; Jones et al., 2009). Similar non-linear complex behaviors are also observed in soft tissues (Fung and Skalak, 1982). Our results showed that PCL-Gel membranes did not have any influence on EV3PB's mechanical performances, so that the device maintains the original values of stiffnesses, stresses, and strains at rupture.

Many studies indicate that appropriate integration of the mesh implant is achieved between 2 and 3 months (Klinge et al., 2002; Boulanger et al., 2006; Morch et al., 2017). After this period, a new cellularized system consisting of implanted mesh, scar tissue, and neo-tissue with stabilized mechanical properties is obtained (Morch et al., 2017). Yet, Plencner et al. were able to shorten the time required for mesh integration down to 6 weeks and improve the wound healing process thanks to a PCL-nanofiber coating of the mesh (Plencner et al., 2014).

Compared to the strategy proposed by Plencner et al., we develop a nanostructured coating combining PCL with a natural polymer, namely Gel. The presence of Gel increases the scaffold biomimicry, which would eventually result in enhanced cellular response and mesh integration (Denis et al., 2015). Indeed, although PCL is a biocompatible polymer, its hydrophobic nature and lack of bioactivity often result in poor tissue integration (Ghasemi-Mobarakeh et al., 2010; Xiang et al., 2018). Oppositely, Gel provides many cell binding sites (e.g., RGD sequence) easily recognized by integrins which to support the adhesion, spreading, proliferation and differentiation of many cell types, such as fibroblasts (Zhang et al., 2005; Gautam et al., 2013; Basar et al., 2017), epithelial cells (Kuppan et al., 2013), hMSCs (Binulal et al., 2014; Jiang et al., 2017), preosteoblasts (Ren et al., 2017), Schwann cells (Gnavi et al., 2015), or myoblasts (Kim et al., 2010). Xiang et al. demonstrated that electrospun PCL scaffolds triggered a severe inflammatory reaction after 2 weeks implantation in rats, whereas the addition of Gel decreased the number of inflammatory cells recruited around the implant, and also promoted the infiltration of host cells (Xiang et al., 2018). Similarly, Gil-Castell et al. proved not only that PCL-Gel nanofibrous membranes did not elicit or sustain undesired inflammatory reactions in rats, but also that they could reduce the scar tissue area after myocardial infarction (Gil-Castell et al., 2020).

In the meshPCL-Gel multicomponent device, the PCL-Gel nanofibrous coating successfully supported the colonization and proliferation of human fibroblasts. The 3D architecture and porosity of the PCL-Gel membrane offered many anchoring points for cell attachment (Sgarminato et al., 2019; Yin et al., 2019) even in the empty pore spaces of the mesh, whose too wide dimensions (Figure 7C) do not favor fast tissue formation. Despite being lower than the control one, metabolic activity of cells seeded onto the meshPCL-Gel device increased throughout the experiment. Particularly, the upswing in metabolic activity from day 3 to day 7 suggests that the reduction of cell number in the first culture days was due to a mild and transient cytostatic effect, rather than to a cytotoxic one. This hypothesis has been further confirmed by the observation that the release of LDH in the culture medium is not increased in case of cells grown on both meshPCL-Gel and PCL-Gel in comparison with control cells. The LDH release is considered a marker of necrotic cell death and, therefore, of cytotoxicity. Indeed, it is released by necrotic cells showing plasma membrane alterations. The fact that in the culture medium of cells grown on either sample the enzymatic activity was significantly lower than that observed in the control cell medium confirms that no cytotoxic effect occurred. This statement has been further confirmed by the observation that no detached cells were present in the culture medium of all samples, as evidenced by counting cells in the Burker chamber. These results are consistent with those reported by Klinge et al. in their *in vivo* study, where the number of fibroblasts at the wound site started to increase from day 3 (Klinge et al., 2002). However, after 21 days cells were able to colonize only a small percentage (~20%) of the mesh area, compared to the control group (~60%).

Further tests are required to investigate the colonization ability of fibroblasts onto the meshPCL-Gel device for longer culturing times, also evaluating their collagen deposition capabilities. When implanted *in vivo*, the nanostructured coating of the meshPCL-Gel device is also expected to improve the host response by presenting a more cell-friendly environment able to modulate inflammation by reducing the FBR (Junge et al., 2012; Wolf et al., 2014). Hence, a characterization of the inflammatory reaction could be conducted both *in vitro in vivo* to evaluate macrophages activity and pro- or anti-inflammatory cytokines release.

Thus, PCL-Gel nanofibrous membrane represents a valuable strategy for the development of a multicomponent hernia mesh device in which it would act as nanostructured coating able to ameliorate cellular response (Eskandarinia et al., 2020), both at the nanoscale and microscale (Denis et al., 2015; Sgarminato et al., 2019), mitigating the immune response, shortening mesh integration times, and ameliorating the wound healing process.

## Conclusions

Surgical meshes employed in abdominal hernia repair often suffer from poor integration with the surrounding tissues, causing patients a state of discomfort and pain. The aim of this study was to develop a novel multicomponent hernia mesh device able to stimulate physiological tissue remodeling during wound healing, thus increasing mesh integration. The multicomponent hernia mesh device was successfully obtained by coating a commercial PP hernia mesh with a nanostructured membrane electrospun from a PCL-Gel blend. First, PCL, Gel, and PCL-Gel nanofibrous membranes were produced at room temperature and characterized through morphological, thermal, physico-chemical, and mechanical analyses. PCL-Gel nanofibers showed a homogeneous and ultrathin morphology, with adequate wettability and appropriate resistance to hydrolytic and enzymatic degradation. Secondly, the biocompatibility of the multicomponent hernia mesh device was assessed with human fibroblast. The nanostructured coating did not induce any cytotoxic effect, rather it was able to support an appropriate cellular response, demonstrating its potentiality as a novel solution for favoring a more prompt and adequate integration of the mesh at the implant site after abdominal wall hernia repair surgeries.

## Data Availability Statement

The raw data supporting the conclusions of this article will be made available by the authors, without undue reservation.

## Author Contributions

IC, CT-T, AG, SC, and MB contributed to the design and implementation of the research. IC, GG, and CT-T carried out the experiments and analyzed the data concerning the multicomponent device. GM and CA carried out the experiments and analyzed the data concerning the biological evaluations. GG and IC wrote the manuscript with the contribution of GM and CA. GC, IC, and CT-T supervised the project and revised the manuscript. All authors discussed the results and contributed to the final manuscript.

## Conflict of Interest

AG, SC, and MB were employed by Dipro Medical Devices S.r.l. The remaining authors declare that the research was conducted in the absence of any commercial or financial relationships that could be construed as a potential conflict of interest.
