# Supplementary material for: In-vitro Characterization of a Hernia Mesh Featuring a Nanostructured Coating
Source: Front Bioeng Biotechnol. 2021 Jan 20;8:589223. doi: 10.3389/fbioe.2020.589223 (PMC7856147; doi:10.3389/fbioe.2020.589223)
Supplement: Supplementary file 1 [file Data_Sheet_1.PDF]

Supplementary information

Preparation of the multicomponent hernia mesh device meshPCL-Gel

Evolution Blue PP meshes were first precoated by dipping them in a 1% wt/v PCL solution in CHL for 10 minutes. After the complete solvent evaporation at room temperature, precoated meshes were attached to the collector with a conductive tape for the deposition of PCL-Gel nanofibers. **Figure 1S** reports the protocol scheme of the steps involved in the fabrication of the meshPCL-Gel device.

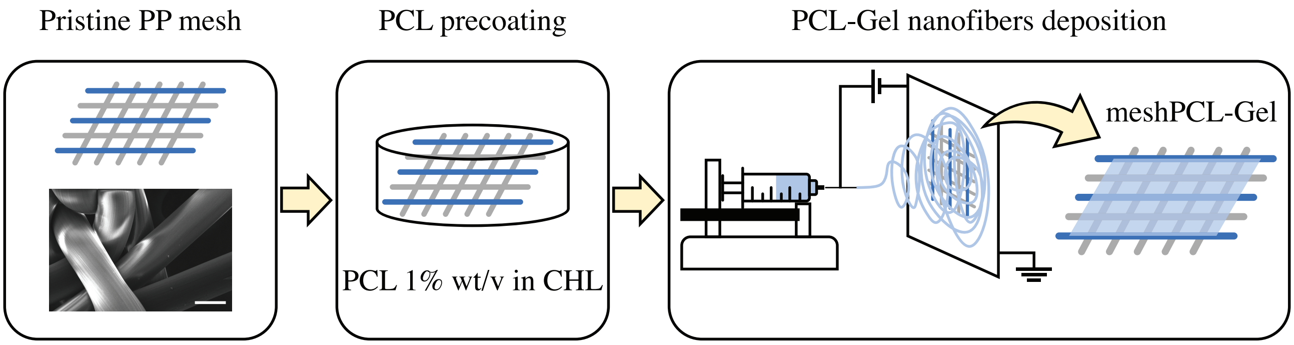

Figure 1S: Schematic of the multicomponent hernia mesh fabrication steps.

PCL-Gel membranes adhesiveness

A qualitative evaluation of PCL-Gel membranes adhesiveness to the PCL-precoated mesh was carried out by gently bending and rubbing samples previously submerged in PBS at room temperature for 40 minutes.

PCL-Gel membranes firmly adhered to the mesh also after immersion of meshPCL-Gel samples in PBS for 40 minutes, with no signs of delamination or separation between the nanofibrous membrane and the underlying mesh, even after bending the sample, or rubbing the deposited membrane with gloves (**Figure 2S, Video 1**).

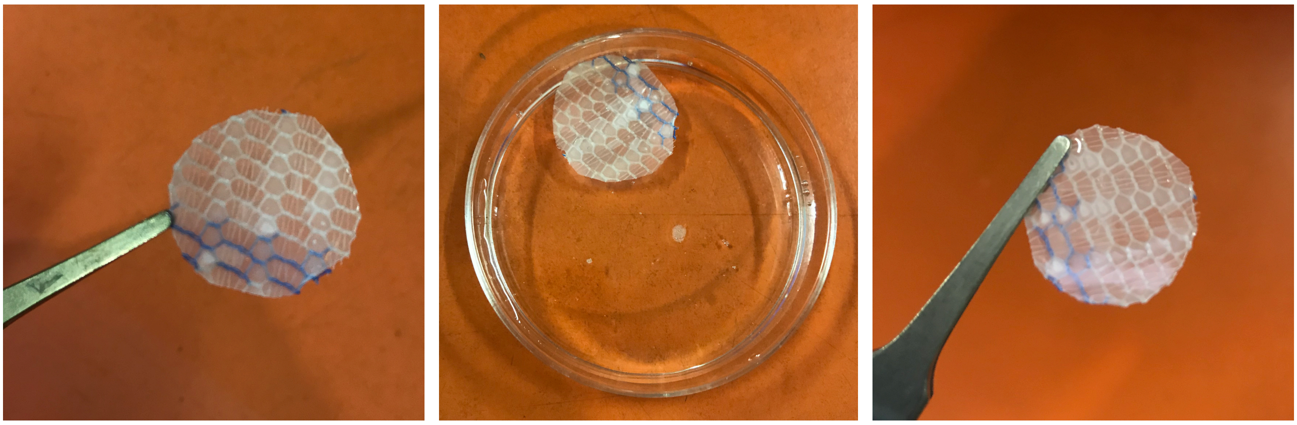

(A) (B) (C)

Figure 2S: Images of the meshPCL-Gel device a) before, b) during, and c) after 40 minutes immersion in PBS.

Fibroblasts colonization ability

Inverted microscope images obtained during MTT test further confirm the ability of human BJ cells in colonizing and growing on meshPCL-Gel and PCL-Gel (**Figure 3S**). In fact, the purple granules evident in the pictures indicate the metabolic activity of mitochondria in viable cells

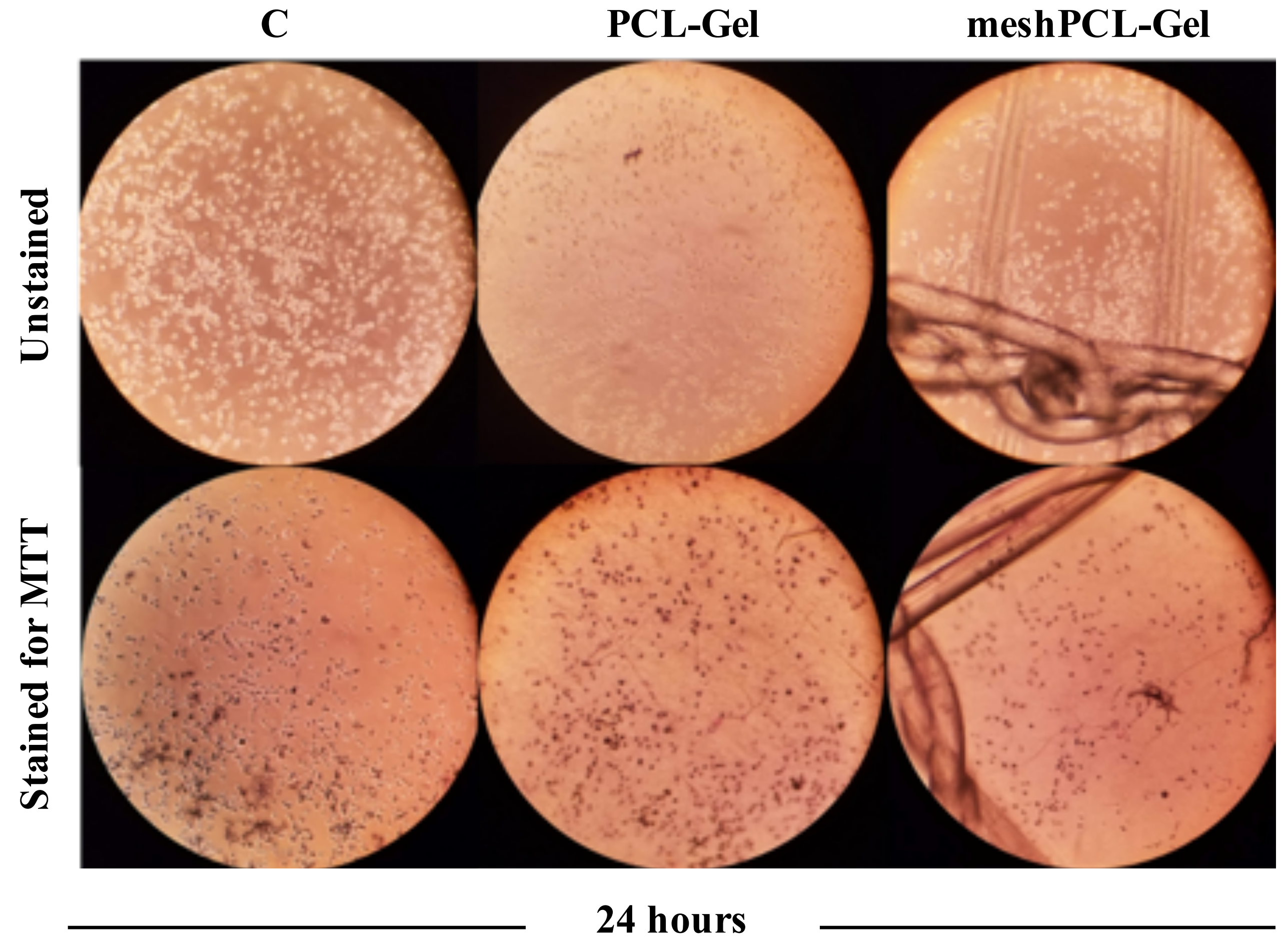

Figure 3S: Inverted microscope images of MTT test performed on BJ human fibroblasts seeded onto the substrates tested.
